# Weight loss effects from vegetable intake: a 12-month randomised controlled trial

**DOI:** 10.1038/ejcn.2014.39

**Published:** 2014-03-26

**Authors:** L C Tapsell, M J Batterham, R L Thorne, J E O'Shea, S J Grafenauer, Y C Probst

**Affiliations:** 1 School of Medicine, Smart Foods Centre, Illawarra Health and Medical Research Institute, University of Wollongong, Wollongong, New South Wales, Australia

## Abstract

**Background/Objectives:**

Direct evidence for the effects of vegetable intake on weight loss is qualified. The study aimed to assess the effect of higher vegetable consumption on weight loss.

**Subjects/Methods:**

A single blind parallel controlled trial was conducted with 120 overweight adults (mean body mass index=29.98 kg/m^2^) randomised to two energy deficit healthy diet advice groups differing only by doubling the serving (portion) sizes of vegetables in the comparator group. Data were analysed as intention-to-treat using a linear mixed model. Spearmans rho bivariate was used to explore relationships between percentage energy from vegetables and weight loss.

**Results:**

After 12 months, the study sample lost 6.5±5.2 kg (*P*<0.001 time) with no difference between groups (*P*>0.05 interaction). Both groups increased vegetable intake and lost weight in the first 3 months, and the change in weight was significantly correlated with higher proportions of energy consumed as vegetables (rho=–0.217, *P*=0.024). Fasting glucose, insulin and triglyceride levels decreased (*P*<0.001 time) and high-density lipoprotein cholesterol levels increased (*P*<0.001 time), with no difference between groups. Weight loss was sustained for 12 months by both groups, but the comparator group reported greater hunger satisfaction (*P*=0.005).

**Conclusions:**

Advice to consume a healthy low-energy diet leads to sustained weight loss, with reductions in cardiovascular disease risk factors regardless of an emphasis on more vegetables. In the short term, consuming a higher proportion of the dietary energy as vegetables may support a greater weight loss and the dietary pattern appears sustainable.

## Introduction

Weight loss occurs when energy intake is less than energy expenditure,^1^ and achieving an energy deficit remains the main dietary target.^2,3^ The evidence for the specific impact of vegetables is qualified. The 2013 Australian Dietary Guidelines (ADG) review found suggestive evidence that vegetable consumption is associated with a reduced risk of weight gain^4^ and the 2010 Dietary Guidelines for Americans found the association was modest, but may be significant in the long term.^5^ These positions may reflect difficulties with food-based dietary trials.^6,7^ Randomised controlled trials provide the appropriate basis for nutrition recommendations,^8^ but for demonstrating long-term effects there are problematic issues. For example, dietary patterns may end up similar in control and intervention groups, or reduced energy intake may override effects from dietary patterns *per se*.

In a recent review, we found that randomised controlled trials demonstrated a greater weight loss from high vegetable intake when it occurred as part of a healthy background diet, the control diet constituted ‘usual intake' and behavioural support was provided.^9^ In such circumstances, it is difficult to attribute the effects to vegetable consumption alone. Shorter feeding studies under highly controlled conditions can provide ‘proof of concept',^8^ but in weight management long-term effects are more clinically relevant. This situation raises a number of questions for how to conduct the research itself.^9^ Some intervention studies have reported beneficial associations between actual vegetable consumption and weight loss for the whole study sample.^10,11^

The classification of vegetables (for example, potato chips vs other vegetables),^12^ gender and behavioural factors,^13^ and background cuisine^14^ also may be important considerations. Low-fat diets have appeared superior to high vegetable diets,^15^ but not always.^16^ Nevertheless, low energy density and increased satiety are plausible reasons for the reduced risk of weight gain with high vegetable intake,^17^ and emerging research suggests the phytochemical composition may be beneficial.^18^ Eating more vegetables may help shift cuisine patterns, proving efficacious in the long term. The aim of this study was to demonstrate the effects of a higher vegetable consumption on sustained weight loss in healthy overweight adults.

## Materials and methods

### Trial design

A single blind parallel randomised controlled trial was conducted between 2010 and 2012 in Wollongong, NSW, Australia. Participants were recruited by advertising in the local media. Inclusion criteria were healthy adults 18–65 years with a body mass index 25–35 kg/m^2^. Exclusion factors were major illnesses, diabetes mellitus, thyroid abnormalities, heavy alcohol consumption, recent acute or chronic disease, changing medications affect weight, weight loss >5 kg in last 3 months, fluctuating exercise patterns, strenuous exercise >1 h per day, pregnancy or lactation, dietary limitations, and dislike of vegetables. One hundred subjects were considered sufficient to detect a minimum between-group weight loss difference of 2.7 kg as significant with 80% power and a two-tailed *α* of 0.05.^19^ This assumes a 40% post-randomisation dropout rate (20 subjects per group) and a within-group weight loss s.d. of 3.5 kg (using available literature^19^). A researcher not associated with the clinical interface (MJB) conducted the randomisation using the RALLOC command in STATA V10.0 (College Station, TX, USA) with the randomisation performed in blocks of 2, 4 or 6 and block sizes randomised within four strata, used to divide the sample by sex and body mass index (25–30 kg/m^2^ and 30–35 kg/m^2^). The master list was provided by strata and reference to the block size and order was removed, and consecutive numbers for allocation provided.

### Dietary intervention

An accredited practising dietitian provided participants with a personalised diet prescription based on core food groups from the Australian Guide to Healthy Eating,^20^ that is, vegetables, fruit, grain foods, meat/fish/eggs/cheese, milk/yoghurt and nuts/seeds/spreads/oils, providing ~80% energy requirements for age, weight and sex as per the Mifflin equation.^21^ The energy intake of the diets was managed by careful dietary modelling of all food groups including vegetables. All participants were requested to consume at least five servings of vegetables each day, but the servings were different between control vs comparator (0.5 vs 1.0 cup cooked; 1 vs 2.0 cups of raw, respectively). Doubling portion size has been shown to be effective in increasing vegetable consumption in a disguised way.^22^ Foods high in saturated fat and added sugars (cakes, biscuits and soft drinks) were discouraged, in keeping with the ADG including the 2013 update.^17^

Initial consultations lasted 1 h, with 30-min follow-up at months 1, 2, 3, 6, 9 and 12 by the same dietitian. E-mail messages were sent 2 weeks before clinic visits. Short message service was sent to participants' mobile phones with reminders of appointments and encouragement to maintain study requirements. Booklets outlining the recommended number of servings of food groups per day and a 4-day estimated food record (including one weekend day) were provided. The high vegetable group were given extra support and materials on use of vegetables.

### Outcome measures

The primary outcome of body weight (kg) was measured in an upright position in minimal clothing and without shoes using scales with a bio-electrical impedance component to also estimate body fat (%) (Tanita TBF-662, Wedderburn Pty Ltd, Ingleburn, NSW, Australia). Body weight was assessed at similar times of the day at 0-, 1-, 2-, 3-, 6-, 9- and 12-month time points.

Secondary clinical outcome measures were indicators of lifestyle disease risk: fasting insulin, glucose and blood lipids: total cholesterol, low-density lipoprotein cholesterol, high-density lipoprotein cholesterol and triglycerides (sampled at 0, 3, 6, 9 and 12 months). Fasting blood samples were sent to quality assured pathology laboratories (Southern Illawarra Medical Laboratory (a fully owned subsidiary of Sonic Health Care Limited, Wollongong, NSW, Australia) for lipids; and Cardinal Bio-Research Pty Ltd, Brisbane, QLD, Australia, for F2 isoprostanes).

Additional information was collected via questionnaire at the 0-, 3-, 6-, 9- and 12-month appointments. The following assessments were also made:

1. Physical activity using the Baecke physical activity questionnaire.^23^
2. Subjective ratings of food intake behaviour using visual analogue scales (found reliable for this purpose,^24,25^ scale completely coinciding with the 4th day of the 4-day food record kept between appointments).
3. Digestive comfort (items relating to thirst, nausea, diarrhoea or constipation using a reference period of the 24 h before the appointment) using a scale (100 mm) within the range of 0–10 (0=not at all, 10=extreme).
4. General diet acceptability score (referencing the period from the last clinical time, scored on a scale of 0–10 (0=extremely unacceptable, 10=extremely acceptable), and including items related to satisfaction with the diet).
5. Perceptions of health, measured with questions from the Medical Outcomes Study 36-item Short Form Health Survey (SF-36): (‘In general, would you say your health is excellent/very good/good/fair/poor? Compared to one year ago, how would you rate your health in general? (better/somewhat better/about the same/somewhat worse/much worse). During the past 4 weeks, to what extent has your physical health or emotional problems interfered with your normal social activities with family, friends, neighbours or groups? (not at all/slightly/moderately/quite a bit/extremely)').

### Compliance

Dietary intake was assessed by diet history interview conducted by an Accredited Practising Dietitian for each participant at 0, 3, 6, 9 and 12 months.^26^ Participants were asked to report their usual intakes of types and amounts of foods starting with the first meal of the day and indicating variations within a 2- to 4-week period. They were asked specific questions on vegetable intake, including identifying the individual vegetables in dishes. Plasma F2 isoprostanes (sampled at 0, 3 and 12 months, see section *Outcome measures*), were measured, given the potential for the phytochemicals in vegetables to act as anti-oxidants, and the previously observed negative associations with F2 isoprostanes and high vegetable diet patterns.^27^

### Data analysis

Data were entered into OpenClinica version beta 3.1.2 clinical trial software (Isovera Inc, Boston, MA, USA) for clinical data management, using the double-entry method by at least two independent researchers for completeness. Dietary data were calculated and analysed using FoodWorks (Version 6; Xyris Pty Ltd, Kenmore Hills, QLD, Australia, 2009) nutrient analysis software using the AUSNUT 2007 food composition survey database.^28^ As weight loss is predicated on total energy intake, vegetable intake data were presented as a percentage of total energy intake.

To examine diet acceptability and monitor changes in appetite, scores were presented so that: for digestive discomfort, the lower the score the less thirst, nausea, diarrhoea or constipation; for satiety, the higher the score the less hungry, less satisfied, lesser sense of fullness, less desire to eat more, and seldom wanting salty, sweet, savoury or fatty foods, respectively; for general diet acceptability, the higher the score the more satisfied, greater ease with preparing food, the greater the effort to adhere to diet plan, more acceptance of core food items and greater ease with continuing on the diet.

Data were analysed using SPSS (version 19.0, SPSS Chicago, IL, USA, 2010) statistical analysis software. Primary and secondary analyses of all continuous variables were conducted using a linear mixed model, which uses all available data regardless of whether the subjects complete the study, the type III fixed effects were used to determine significance. Skewed variables were log_e_ or square root transformed before analysis, and values before transformation were reported to assist with interpretations. The analysis was conducted on an intention-to-treat basis on trial completion. Owing to non-normal distributions, Spearman's correlations were used to assess relationships between changes in vegetable intake and isoprostane levels and to assess correlations between change in weight and change in vegetable consumption (% energy). The study was approved by the University of Wollongong Human Research Ethics Committee and registered with Australia New Zealand Clinical Trials Register Network (ACTRN12610000784011).

## Results

### Sample

**Figure 1:**
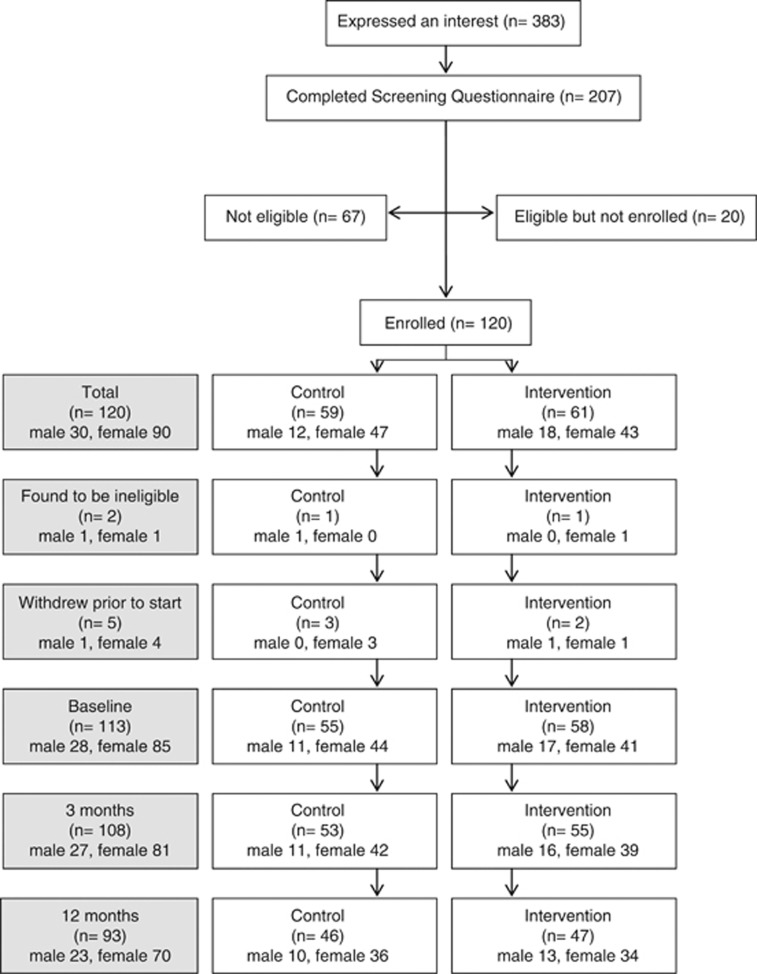
Enrolment, randomisation and follow-up of study participants.

Of the 383 adults who volunteered for the study, 207 completed screening. Of these 67 did not meet eligibility criteria and 20 did not enrol. Thus, *n*=120 adults were randomised to control and comparator groups and *n*=93 completed through to 12 months (77.5% completion; Figure 1). The mean age was 48.9±9.3 years. Seven participants withdrew before the start and a further *n*=18 participants later withdrew consent because of moving out of the area, family or personal health issues. Data from *n*=2 participants were not included in the final analysis because of extreme non-compliance and starting medications likely to affect weight (data removed after 3 months, Figure 1). There were no adverse effects from the trial.

### Between-group effects

**Figure 2:**
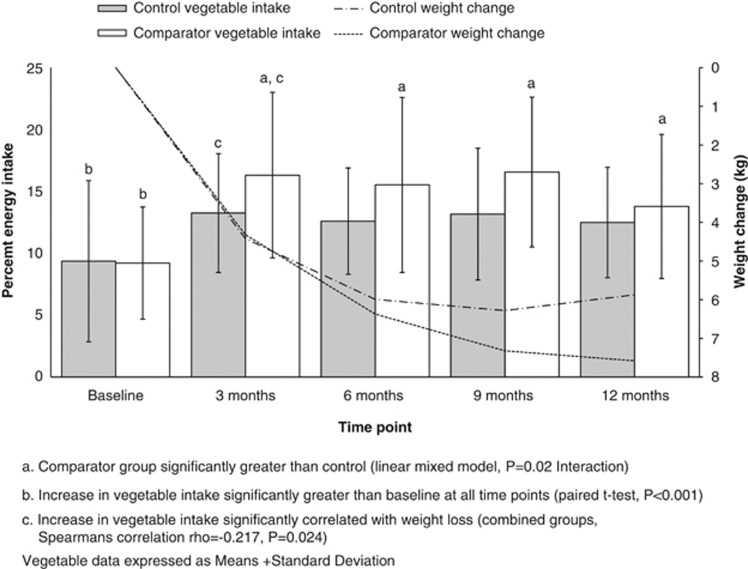
Vegetable intake expressed as a proportion of total energy intake from baseline to 12 months.

After 12 months, both groups lost weight (*P*<0.001, time effect), with no difference between groups in weight loss (*P*=0.776, interaction effect; Table 1) or reported energy intake (*P*=0.701, interaction effect; Table 2). Reported physical activity was not different between groups and did not change throughout the trial (*P*=0.170, time; *P*=0.690, interaction; Baecke score). Compared with the controls, the comparator group reported consuming a significantly greater proportion of energy intake from vegetables (*P*=0.020, interaction; Figure 2).

### Intervention effects

The sample produced a mean weight loss of 6.5±5.2 kg (range –27.8 to +5 kg) and this was associated with reported change in energy intake (*P*=0.002, 3 months; *P*=0.009 12 months) estimated at about –2000 kJ/day (*P*<0.001, time effect; Table 2). Reported energy intake was significantly lower than baseline at all-time points (*P*<0.001). Vegetable consumption increased substantially in the first 3 months (Table 2) and the proportion of energy consumed as vegetables was significantly different from baseline at all-time points (*P*<0.001; Figure 2). Both groups reduced energy intake from high-energy-dense vegetables (time effect *P*=0.005) and increased energy intake from low-energy-dense vegetables (*P*<0.001; Table 2). Most of the weight loss occurred in the first 3 months and was maintained. By 12 months, there was a shift in reported consumption away from higher energy vegetables (*P*=0.005, time effect) to lower energy vegetables (*P*<0.001, time effect).

**Figure 3:**
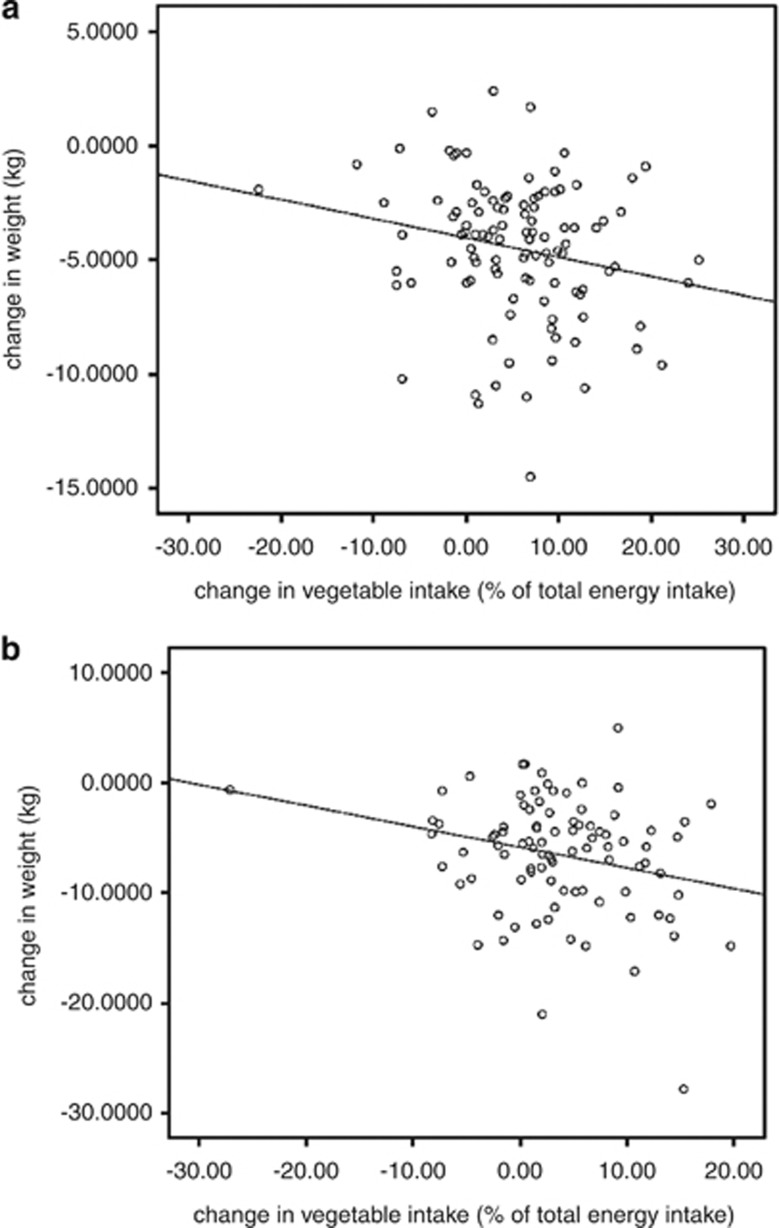
(**a**) Relationship between change in weight and change in vegetable intake as % of total energy intake at 3 months. (**b**) Relationship between change in weight and change in vegetable intake as % of total energy intake at 3 months.

The change in weight was significantly correlated with the increase in proportion of energy consumed as vegetables at 3 months (rho=–0.217; *P*=0.024; *n*=108; Figure 3a). The association did not remain significant at 12 months (rho=–0.193; =0.06; *n*=92; Figure 3b). With increasing vegetable intake, there were reductions in isoprostane levels and this was significant for change in vegetable intake from baseline to 3 months represented as percentage of dietary energy (rho=–0.198; *P*=0.046) and from baseline to 12 months (rho=–0.231; *P*=0.030). There were significant changes in macronutrient intakes: a reduction in energy from total fat by about 5%, an increase in protein energy by about 3% and an increase in carbohydrate energy by about 2% (time effects all *P*<0.000; data not shown).

### Secondary effects

All biochemical parameters improved with the exception of the near normal total and low-density lipoprotein cholesterol levels. Total high-density lipoprotein increased, improving the total cholesterol: high-density lipoprotein ratio (Table 3). Diet acceptability was high throughout the trial (Table 4). All digestive discomfort scores remained relatively low. Ratings for constipation, diarrhoea and nausea were low (range 0.59±1.39–1.71±3.07, maximum 10) with the natural sensation of thirst slightly higher (range 3.32+2.51–4.44±2.86, maximum 10).

The higher vegetable group reported a greater increase in hunger satisfaction (*P*=0.005 interaction) with a marginally significant decrease in score by the control group from 6 to 12 months (*P*=0.077). All trial participants reported being less hungry throughout the trial (*P*<0.001, time), they were less inclined to eat more food (*P*=0.002, time) and to desire sweet foods (*P*<0.001; 0.049, time, respectively). They reported less desire for fatty food (*P*=0.001, time).

All items on general diet acceptability were high (around 7–8 where 10=extremely acceptable), with no differences between groups (*P*>0.05, interaction), except ‘ease to continue' item (*P*=0.050, interaction), which was lower score for the higher vegetable group at 6 months (*P*=0.012, time) only.

The study participants' perceptions of personal health appeared to improve between baseline and 12 months (data not shown). The proportion reporting their health as ‘excellent' increased fourfold and the proportion noting ‘very good' doubled compared with the baseline. About half of the trial participants reported their health as being ‘better than a year ago'. The proportion indicating that physical or emotional problems interfered with normal activities decreased three- to fourfold.

## Discussion

Our finding that low-energy healthy dietary advice^17^ produced substantial and sustained weight loss regardless of differences in advice on vegetables (Table 1) is consistent with other studies of a similar nature.^15,16,29^ However, by examining the variation in *actual* vegetable intake across the study sample, we confirmed similar reported findings^11^ that vegetable intake correlated with weight loss (Figure 3). The shifts in macronutrient profile were consistent with an increase in the relative amount of vegetables in a dietary pattern comprising core staple foods, as was advised. The secondary analysis warrants further discussion because, despite the reported value of increasing vegetable portion size,^22^ this advice strategy did not prove sufficient to create a difference in energy intakes in our groups receiving healthy dietary advice based on 80% of energy requirements.

The primary analysis confirms that total dietary energy is the most important dietary variable for weight loss,^2^ but does little to clarify on best ways to get there and why. We know from studies using *ad libitum* dietary approaches that supporting an increased vegetable intake does not reduce energy intake,^30^ or compare favourably against a low-kilojoule diet,^11^ (and there are challenges in encouraging greater consumption of vegetables).^31^ Low-kilojoule/low-fat dietary advice appears to be more sustainable than diets focusing on vegetables intake,^32^ although providing vegetables in the short term may enable what is known as ‘proof of concept' conditions.^8^ We need to look beyond the single outcome measure of weight loss. Although we did achieve significant differences between groups in energy consumed as vegetables, this was not enough to have an effect on weight loss (Figure 2) because they were both reducing energy intake. For the group, however, vegetable intake was correlated with weight loss, as has been found before.^11^ Indeed a greater number of participants or a longer period of follow-up may have proven more informative. Nevertheless, as energy balance is a total diet effect, any single food group can only make a contribution to weight loss. Weight maintenance is the key goal and this requires sustained dietary change. Our data suggest increasing the relative energy contribution of vegetables may provide a viable key strategy, and we explored this further from a broader perspective.

The comparator group reported greater hunger satisfaction (*P*=0.005), consistent with reports suggesting that vegetables act via controlling for hunger.^16^ Reviews suggest that encouraging and supporting healthy diets with significant amounts of vegetables are likely to be effective because, although there are limitations on other foods, the central message is positive.^33^ In terms of the vegetable content of both diets, the literature suggests that establishing long-term habituation through repeat presentation of vegetables at all meals^34^ and the encouragement of vegetable variety^35,36^ may help establish lasting change essential to prevent weight regain,^37^ and we appeared to be seeing that. It should also be noted that the dietary advice strategy focused on shifting from high- to low-energy-dense vegetables and this was reflected in the reported dietary change for the study sample (Table 2).

There were other health considerations. The biomarker (F2 isoprostane) results were consistent with observations that a high vegetable intake is associated with a lower level of the markers of oxidative damage.^27^ The pattern of reduction in F2 isoprostanes was consistent with the pattern of weight change and vegetable consumption. A recent review of vegetables containing phytochemicals with potential anti-obesity properties specifically identifies common vegetables as sources of known agents that may have contributed to these effects.^18^ Although this was not a particularly high-risk sample, the participants were tending to insulin resistance, yet they showed significant reductions in waist circumference (Table 2), fasting insulin and triglyceride levels and increases in high-density lipoprotein cholesterol (Table 3). These changes are indicative of a substantial risk reduction for developing type 2 diabetes.^38^ The effects may not be limited to weight loss alone and this area warrants further research.

There were necessary controls on energy intakes and the background diet in this study, which made it difficult to expose effects of a single food group, as acknowledged in the literature.^7^ Dietary change is not just about desirable foods going into the diet; it is also about taking others out. In a recent publication,^39^ we reported that participants with poor baseline dietary patterns lose substantially more weight than those starting with healthy dietary patterns. Future research might test whether shifting eating patterns from a low to a high vegetable intake may have an impact on achieving weight loss by replacing poor quality foods in the total diet.

In conclusion, a dietary energy deficit can be achieved in any number of ways.^3^ Advice to consume a healthy low-energy diet including five servings of vegetables per day can lead to sustained weight loss, with associated reductions in cardiovascular disease risk factors. In the short term, consuming a higher proportion of the dietary energy as vegetables may support a greater weight loss and the dietary pattern appears sustainable.

## Figures and Tables

**Table 1:** Anthropometric measures from baseline to 12 months (mean ±s.d.)

*Variable*	*Baseline*		*3 Months*		*6 Months*		*9 Months*		*12 Months*		P*-values*^a^		
	*Control (*n*=55)*	*Comparator (*n*=58)*	*Control (*n*=53)*	*Comparator (*n*=55)*	*Control (*n*=50)*	*Comparator (*n*=47)*	*Control (*n*=43)*	*Comparator (*n*=46)*	*Control (*n*=46)*	*Comparator (*n*=47)*	*Time*	*Group*	*Interaction*
Males|females	11|44	17|41	11|42	16|39	9|41	14|33	8|35	13|34	10|36	13|47			
Weight(kg)	84.89±9.86	84.60±13.05	80.47±9.75	80.28±12.87	78.90±8.98	78.23±11.25	78.61±9.25	77.28±10.95	79.02±9.32	77.02±10.74	<0.001	0.949	0.776
BMI(kg/m^2^)	29.84±2.57	30.11±2.89	28.19±2.50	28.58±2.80	27.71±2.30	27.73±2.51	27.82±2.40	27.52±2.42	27.79±2.35	27.51±2.57	<0.001	0.657	0.763
Body fat (%)^b^	40.5 (37.50–43.00)	38.55 (33.90–42.82)	39.30 (33.00–43.00)	38.00 (31.40–41.70)	39.35 (33.60–42.12)^c^	37.10 (29.92–40.40)^d^	38.60 (33.10–42.30)	36.60 (30.05–40.20)^e^	38.10 (32.35–41.25)	36.75 (28.77–40.45)^d^	<0.001^f^	0.280^f^	0.615^f^
Waist (cm)	98.48±9.39^g^	97.44±9.32	94.66±8.86^h^	94.05±9.29	93.95±8.25^i^	90.28±15.02^d^	92.84±8.43^j^	91.37±8.89^k^	92.80±8.13^l^	90.77±9.40^e^	<0.001	0.489	0.451
Hip (cm)	108.56±7.24^g^	109.34±7.64	104.46±7.30^h^	105.23±8 .29	104.73±7.03^i^	103.37±7.83^d^	103.88±6.48^j^	103.40±8.41^k^	103.74±6.79^l^	103.30±8.47^e^	<0.001	0.621	0.560

Abbreviations: BMI, body mass index; IQR, interquartilerange.

a Linear mixed model, significant at *P*<0.05.

b Median(IQR).

c *n*=48.

d n=46.

e *n*=45.

f Log transformed.

g *n*=54.

h *n*=52.

i *n*=47.

j *n*=38.

k *n*=43.

l *n*=44.

**Table 2:** Reported energy intake, physical activity score and reported vegetable intakes from baseline to 12 months (mean ±s.d.)

*Variable*	*Baseline*		*3 Months*		*6 Months*		*9 Months*		*12 Months*		P*-values*^a^		
	*Control (n=55)*	*Comparator (n=58)*	*Control (n=53)*	*Comparator (n=56)*	*Control (n=50)*	*Comparator (n=47)*	*Control (n=43)*	*Comparator (n=46)*	*Control (n=45)*	*Comparator (n=47)*	*Time*	*Group*	*Interaction*
Energy intake (kJ)	8905.08 ±2414.84	8895.72 ±2282.54	6182.96 ±1479.94	6296.86 ±1252.04	6247.67 ±1363.47	6390.99±1429.40	6468.63±1345.19	6324.31±1046.15	6492.42±1662.03	6654.41±1152.94	<0.001	0.483	0.701
Physical activity (Baecke)	7.61±1.36	7.58±1.42	7.70±1.27	7.67±1.32	7.83±1.31	7.56±1.25	7.66±1.36	7.81±1.23	7.81±1.47^b^	7.77±1.24	0.17	0.32	0.69
Total vegetable intake (kJ)	785.01±477.66	792.78±384.87	785.34±257.38	985.32±368.99	759.24±238.71	956.63±402.80	844.88±294.90	840.63±373.05	775.12±247.13	888.44±342.33	0.187	0.027	0.025
High-energy-dense vegetables (kJ)^c^	464.79±381.13	471.91±322.77	296.74±185.90	462.54±265.33	267.21±196.36	433.25±280.08	371.55±243.59	381.73±244.89	333.03±199.54	417.84±234.87	0.005	0.005	0.30
Low-energy-dense vegetables (kJ)	320.23±155.85	320.87±172.15	488.61±206.15	522.78±230.62	492.03±220.77	523.39±260.75	473.33±206.87	449.14±229.18	442.09±140.02	460.79±228.00	<0.001	0.872	0.630
% Dietary energy from vegetables^b^	9.36±6.51	9.21±4.53	13.25±4.80	16.31±6.69	12.60±4.30	15.53±7.07	13.17±5.32	16.57±6.05	12.49±4.46	13.79±5.82	<0.001	0.086	0.020

a Linear mixed model, significant at *P*<0.05.

b Percentage kJ value of total veg divided by total kJ value from diet history interview.

c Typical high-energy-dense vegetables were selected for this category—avocado, potato, sweet potato, legumes and sweet corn. Pumpkin has a similar energy density to beetroot and onions and therefore was not included in this group.

**Table 3:** Clinical variables from baseline to 12 months (mean ±s.d.)

*Variable*	*Baseline*		*3 Months*		*6 Months*		*9 Months*		*12 Months*		P*-values*^a^		
	*Control (n=54)*	*Comparator (n=58)*	*Control (n=52)*	*Comparator (n=55)*	*Control (n=50)*	*Comparator (n=47)*	*Control (n=43)*	*Comparator (n=46)*	*Control (n=46)*	*Comparator (n=47)*	*Time*	*Group*	*Interaction*
Males/females	11/43	17/41	11/41	16/39	9/41	14/33	8/35	13/33	10/36	13/47			
Glucose (mmol/l)	5.23±0.53	5.31±0.50	5.10±0.39	5.17±0.42	5.20±0.38	5.23±0.46	5.15±0.40	5.08±0.45	5.17±0.36	5.20±0.37	0.001	0.747	0.652
Insulin (m/Ul)^b^	11.35 (8.33–15.10)	10.70 (7.45–13.65)	9.40 (6.83–12.28)	8.30 (6.30–10.70)	9.75 (6.50–12.25)	8.60 (6.30–10.50)	8.40 (6.80–13.10)	7.75 (5.98–10.70)	8.80 (6.15–11.90)	8.50 (6.10–10.90)	<0.001^c^	0.093^c^	0.675^c^
Tot Chol (mmol/l)	5.21±0.88	5.20±0.88	5.01±0.83	5.08±0.79	5.22±0.74	5.26±0.77	5.20±0.81	5.13±0.74	5.22±0.98	5.20±0.80	0.202	0.914	0.778
Trigs (mmol/l)^b^	1.24 (0.80–1.59)	1.02 (0.75–1.45)	1.01 (0.77–1.40)	1.07 (0.82–1.30)	0.95 (0.81–1.36)	0.99 (0.79–1.31)	0.92 (0.75–1.53)	0.90 (0.73–1.26)	0.97 (0.75–1.46)	0.94 (0.71–1.17)	<0.001^d^	0.469^d^	0.749^d^
HDL (mmol/l)^b^	1.34 (1.14–1.67)	1.40 (1.16–1.62)	1.38 (1.18–1.55)	1.29 (1.17–1.52)	1.56 (1.32–1.73)	1.40 (1.25–1.64)	1.56 (1.36–1.73)	1.51 (1.29–1.70)	1.51 (1.36–1.70)	1.46 (1.26–1.72)	<0.001^c^	0.429^c^	0.373^c^
LDL (mmol/l)	3.19±0.80	3.25±0.82	3.10±0.74	3.20±0.71	3.13±0.69	3.29±0.67	3.12±0.73	3.14±0.68	3.14±0.84	3.20±0.71	0.163	0.533	0.702
Tot Chol: HDL	3.90±1.16	3.89±1.28	3.79±1.03	3.90±0.90	3.51±0.88	3.74±0.85	3.43±0.81	3.52±0.96	3.46±0.90	3.56±0.92	<0.001	0.563	0.671
Isoprostanes (pg/ml)	181.48±62.54	175.71±59.51	158.50±52.59	141.25±47.47					214.21±77.75	198.95±64.09	<0.001	0.159	0.628

Abbreviations: HDL, high-density lipoprotein; IQR, interquartile range; LDL, low-density lipoprotein; Tot Chol, total cholesterol; Trigs, triglycerides.

a Linear mixed model, significant at *P*<0.05.

b Median (IQR).

c Log transformed.

d Square root transformed.

**Table 4:** Digestive comfort, satiety and diet acceptability ratings from baseline to 12 months (mean ±s.d.)

*Variable*	*Baseline*		*3 Months*		*6 Months*		*9 Months*		*12 Months*		P*-values*^a^		
	*Control (*n*=55)*	*Comparator (*n*=58)*	*Control (*n*=53)*	*Comparator (*n*=56)*	*Control (n=50)*	*Comparator (n=47)*	*Control (n=43)*	*Comparator (n=46)*	*Control (n=46)*	*Comparator (n=47)*	*Time*	*Group*	*Interaction*
*Digestive comfort*													
Thirst (VAS)	4.142.62	4.36±2.33	3.85±2.72	3.75±2.93	4.44±2.86	3.67±2.61	3.35±2.54^b^	3.79±2.67^b^	3.32±2.51^c^	4.09±2.74	0.264	0.823	0.127
Nausea (VAS)	1.64±3.22	1.71±3.07	1.00±2.25	1.05±1.97	1.08±2.36	1.66±2.90	1.367±2.66^b^	1.145±2.21^b^	0.71±1.45	0.89±1.88	0.174	0.471	0.809
Diarrhoeal (VAS)	0.68±1.71	0.59±1.39	0.58±1.40	0.76±1.70	1.05±1.79	0.74±1.60	0.77±1.53	0.93±1.88	0.77±1.28	0.70±1.27	0.409	0.978	0.693
Constipation (VAS)	0.94±1.78	1.26±2.08	1.15±1.83	1.31±2.09	1.61±2.28	1.32±2.08	1.52±2.35	1.64±2.29	1.54±2.36	1.19±1.92	0.169	0.799	0.696

*Satiety scales*													
Hunger (VAS)	5.17±2.24^d^	5.04±2.15	6.39±2.08^d^	6.32±2.28^e^	5.69±2.30^f^	6.35±2.32^g^	5.87±2.09^h^	6.28±2.29^i^	6.29±2.28^g^	6.27±2.22^i^	<0.001	0.460	0.440
Satisfied (VAS)	4.13±2.10^j^	3.90±2.03	4.35±2.02^d^	4.06±2.10^e^	4.72±1.69^b^	3.42±1.63^g^	3.95±1.60^h^	4.14±2.00^i^	3.67±1.80^k^	4.28±2.05^i^	0.827	0.831	0.005
Full (VAS)	4.14±2.60^j^	4.25±2.18	4.26±1.96^l^	3.85±2.36^d^	4.60±2.42^b^	3.79±2.43^g^	4.15±2.09^h^	4.19±2.04^i^	3.69±2.21^k^	3.82±1.92^i^	0.363	0.954	0.383
Eat more (VAS)	3.96±2.61^e^	4.25±2.43	4.72±2.33^d^	5.62±2.50^e^	4.06±2.20^f^	5.54±2.71^g^	4.68±2.32^h^	5.10±2.75^i^	4.47±2.30^k^	5.13±2.43^i^	0.002	0.029	0.245
Sweet (VAS)	3.14±2.31^j^	3.40±2.88	4.63±2.52^d^	4.84±3.07^e^	4.09±2.45^b^	4.96±2.94^g^	4.32±2.34^h^	5.11±2.91^i^	4.69±2.35^k^	4.68±2.82^i^	<0.001	0.445	0.799
Salty (VAS)	4.72±3.06^j^	5.34±2.92	4.86±2.83^d^	6.40±2.68^e^	5.31±2.81^b^	5.92±2.88^g^	5.16±2.80^h^	6.07±2.77^i^	4.95±2.85^k^	6.24±2.68^i^	0.181	0.119	0.171
Savoury (VAS)	3.89±2.45^j^	4.62±2.70	4.68±2.53^d^	5.16±2.60^e^	4.69±2.69^b^	4.99±2.38^r^	4.29±2.27^h^	5.10±2.67^i^	4.51±2.53^k^	5.13±2.75^i^	0.049	0.341	0.654
Fatty (VAS)	6.40±2.71^j^	6.03±2.90	6.59±2.87^d^	7.34±2.61^e^	6.48±2.76^b^	7.31±2.89^g^	6.95±2.53^h^	7.60±2.50^i^	6.68±2.82^k^	7.84±2.23^i^	0.001	0.216	0.084

*Diet acceptability*													
Satisfaction	7.98^m^±1.90^e^	7.52^m^±2.31	7.82±1.99	7.78±1.90^o^	7.94±1.79^n^	7.63±2.33^o^	7.87±1.98^k^	7.70±1.93^h^	8.13±2.29^c^	7.84±2.26	0.786	0.287	0.905
Ease	7.82^m^±2.03^e^	7.04^m^±2.43	7.75±2.23	7.44±2.04^o^	7.65±2.12^n^	7.09±2.53	7.67±2.34^f^	6.85±2.28^f^	8.35±1.81^o^	7.40±2.30	0.093	0.018	0.552
Effort	3.59^m^±2.57^d^	4.45^m^±2.67	3.50±2.59	3.78±2.65^o^	3.06±1.83^p^	4.48±2.97	3.13±2.56^f^	4.36±2.79^q^	3.62±2.79^o^	4.30±2.92	0.521	0.019	0.256
Fruit	7.77^m^±2.28^e^	7.97^m^±2.23	7.70±2.00	7.91±2.05^o^	7.92±1.94^n^	7.86±2.10	8.16±1.82^b^	8.39±1.49^f^	8.12±1.83^o^	7.79±2.17	0.057	0.976	0.348
Vegetables	8.27^m^±1.58^e^	7.79^m^±1.74	8.32±1.39	8.42±1.37^o^	8.35±1.51^n^	8.03±1.94	8.30±1.80^b^	8.20±1.75^f^	8.58±1.60^o^	8.05±1.98	0.133	0.186	0.173
Grains	8.36^m^±1.73^e^	7.99^m^±1.94	7.95±1.71	8.08±1.80^o^	8.09±1.66^n^	7.87±2.14	8.10±1.58^b^	7.80±1.91^f^	8.35±1.37^o^	7.50±2.24	0.738	0.235	0.081
Meat, fish and eggs	6.87^m^±2.73^e^	7.12^m^±2.50	7.10±2.51	7.47±2.29^o^	7.24±2.28^n^	6.81±2.56	7.39±2.29^b^	7.50±2.02^f^	7.48±2.40^o^	6.98±2.52	0.323	0.881	0.114
Milk and Yoghurt	8.02^m^±1.62^e^	7.76^m^±2.07	7.69±1.75	8.13±1.98^o^	7.51±2.04^n^	7.92±2.09	8.15±1.40^b^	7.96±1.90^f^	8.20±1.66^o^	7.90±1.89	0.317	0.905	0.104
Nuts and oils	8.03^m^±1.85^e^	8.00^m^±1.95	7.56±2.01	8.14±1.85^o^	7.96±1.55^n^	7.92±1.99	8.13±1.40^b^	7.93±2.07^f^	8.22±1.47^o^	7.61±2.34	0.651	0.821	0.057
Ease to continue	8.16^m^±1.75^e^	7.45^m^±1.89	7.83±1.76	7.77±1.54^j^	7.81±1.93^n^	6.87±2.26	7.46±2.25^b^	7.35±1.89^f^	7.95±1.89^o^	7.17±2.62	0.035	0.047	0.050

Abbreviation: VAS, visual analogue scale.

Digestive comfort: (0= not at all, 10= extreme) the lower the score, the less thirst, nausea, diarrhoea, constipation. Satiety scales: (0= always, 10= never) higher the score, the less hungry, less satisfied, less totally full, never hungry, seldom wanting salty, sweet, savoury or fatty. Diet acceptability: (0= extremely unacceptable, 10 = extremely acceptable). The higher the score, the more satisfied, ease of preparing food, more acceptable fruit, vegetables, grains, milk, nuts and ease of continuing. The higher the score, the less effort to prepare food.

a Linear mixed model, significant at *P*<0.05.

b *n*=44.

c *n*=45.

d *n*=52.

e *n*=53.

f *n*=43.

g *n*=39.

h *n*=42.

i *n*=35.

j *n*=54.

k *n*=40.

l *n*=51.

m Questionnaire taken at 1 month. *o n*=55.

n *n*=49.

o *n*=46.

p *n*=48.

q *n*=41.

r *n*=38.

## References

[bib1] Hall KD Heymsfield SB Kemnitz JW Klein S Schoeller DA Speakman JR Energy balance and its components: implications for body weight regulation Am J Clin Nutr 2012 95 989 994 22434603 10.3945/ajcn.112.036350 PMC3302369

[bib2] Bray GA Diet and exercise for weight loss JAMA 2012 307 2641 22735436 10.1001/jama.2012.7263

[bib3] Sacks F Bray G Carey V Smith S Ryan D Anton S Comparison of weight-loss diets with different compositions of fat, protein and carbohydrates New Engl J Med 2009 360 859 19246357 10.1056/NEJMoa0804748 PMC2763382

[bib4] National Health and Medical Research Council A review of the evidence to address targeted questions to inform the revision of the Australian Dietary Guidelines-Evidence Statements Ageing DoHa CanberraCommonwealth of Australia 2011

[bib5] Dietary Guidelines Advisory Committee Report of the dietary guidelines advisory committee on the Dietary Guidelines for Americans The Secretary of Agriculture and the Secretary of Health and Human Services Washington, DCU.S. Department of Agriculture, Agricultural Research Service 2010

[bib6] Truswell AS Levels and kinds of evidence for public-health nutrition Lancet 2001 357 1061 1062 11297955 10.1016/S0140-6736(00)04308-7

[bib7] Jacobs DR Tapsell LC Temple NJ Food synergy: the key to balancing the nutrition research effort Public Health Rev 2011 33 1 23

[bib8] Mann JI Morenga LT Diet and diabetes revisited, yet again Am J Clin Nutr 2013 97 453 454 23364020 10.3945/ajcn.112.057547

[bib9] Tapsell LC Dunning A Warensjo E Lyons-Wall P Dehlsen K Effects of vegetable consumption on weight loss: a review of the evidence with implications for design of randomised controlled trials Crit Rev Food Sci Nutr 2014 54 1529 1538 24580555 10.1080/10408398.2011.642029

[bib10] Sartorelli DS Franco LJ Cardoso MA High intake of fruits and vegetables predicts weight loss in Brazilian overweight adults Nutr Res 2008 28 233 238 19083413 10.1016/j.nutres.2008.02.004

[bib11] Whigham LD Valentine AR Johnson LK Zhang Z Atkinson RL Tanumihardjo SA Increased vegetable and fruit consumption during weight loss effort correlates with increased weight and fat loss Nutr Diabetes 2012 2 e48 23449500 10.1038/nutd.2012.22 PMC3488810

[bib12] Mozaffarian D Hao T Rimm EB Willett WC Hu FB Changes in diet and lifestyle and long-term weight gain in women and men New Eng J Med 2011 364 2392 2404 21696306 10.1056/NEJMoa1014296 PMC3151731

[bib13] Vergnaud AC Norat T Romaguera D Mouw T May AM Romieu I Fruit and vegetable consumption and prospective weight change in participants of the European prospective investigation into cancer and nutrition-physical activity, nutrition, alcohol, cessation of smoking, eating out of home, and obesity study Am J Clin Nutr 2012 95 184 193 22170373 10.3945/ajcn.111.019968

[bib14] Ajala O English P Pinkney J Systematic review and meta-analysis of different dietary approaches to the management of type 2 diabetes1-3 Am J Clin Nutr 2013 97 505 516 23364002 10.3945/ajcn.112.042457

[bib15] Lapointe A Weisnagel SJ Provencher V Bégin C Dufour-Bouchard AA Trudeau C Comparison of a dietary intervention promoting high intakes of fruits and vegetables with a low-fat approach: long-term effects on dietary intakes, eating behaviours and body weight in postmenopausal women Br J Nutr 2010 104 1080 1090 20482930 10.1017/S0007114510001716

[bib16] Ello-Martin JA Roe LS Ledikwe JH Beach AM Rolls BJ Dietary energy density in the treatment of obesity: a year-long trial comparing 2 weight-loss diets Am J Clin Nutr 2007 85 1465 1477 17556681 10.1093/ajcn/85.6.1465 PMC2018610

[bib17] National Health and Medical Research Council Australian Dietary Guidelines CanberraNational Health and Medical Research Council 2013

[bib18] Williams DJ Edwards D Hamernig I Jian L James AP Johnson SK Vegetables containing phytochemicals with potential anti-obesity properties: a review Food Res Int 2013 52 323 333

[bib19] Allison DB Gadbury G Schwartz LG Murugesan R Kraker JL Heshka S A novel soy-based meal replacement formula for weight loss among obese individuals: a randomized controlled clinical trial EurJ Clin Nutr 2003 57 514 522 12700612 10.1038/sj.ejcn.1601587

[bib20] NHMRC Australian guide to healthy eating Ageing DoHa CanberraCommonwealth of Australia 1998

[bib21] Mifflin M St Jeor S Hill L Scott B Daugherty SA Koh Y A new predictive equation for testing energy expenditure in healthy individuals Am J Clin Nutr 1990 51 241 247 2305711 10.1093/ajcn/51.2.241

[bib22] Rolls BJ Roe LS Meengs JS Portion size can be used strategically to increase vegetable consumption in adults Am J Clin Nutr 2010 91 913 922 20147467 10.3945/ajcn.2009.28801 PMC2844679

[bib23] Baecke JA Burema J Frijters JE A short questionnaire for the measurement of habitual physical activity in epidemiological studies Am J Clin Nutr 1982 36 936 942 7137077 10.1093/ajcn/36.5.936

[bib24] Flint A Raben A Blundell JE Astrup A Reproducibility, power and validity of visual analogue scales in assessment of appetite sensations in single test meal studies Int J Obesity Relat Metab Disord 2000 24 38 48 10.1038/sj.ijo.0801083 10702749

[bib25] Geiselmann PJ Anderson AM Dowdy ML West DB Redmann SM Smith SR Reliability and validity of a macronutrient self-selection paradigm and a food preference questionnaire Physiol Behav 1998 63 919 928 9618017 10.1016/s0031-9384(97)00542-8

[bib26] Martin GS Tapsell LC Batterham MJ Russell KG Relative validity of a diet history interview in an intervention trial manipulating dietary fat in the management of type 2 diabetes mellitus Prev Med 2003 36 420 428 12649050 10.1016/s0091-7435(02)00054-3

[bib27] Meyer KA Sijtsma FPC Nettleton JA Steffen LM Van Horn L Shikany JM Dietary patterns are associated with plasma F2-isoprostanes in an observational cohort study of adults Free Radical Biol Med 2013 57 201 209 22982044 10.1016/j.freeradbiomed.2012.08.574 PMC3872789

[bib28] Food Standards Australia New Zealand AUSNUT 2007 -Australian Food, Supplement and Nutrient Database for Estimation of Population Nutrient Intakes CanberraFSANZ 2008

[bib29] Lapointe A Weisnagel SJ Provencher V Bégin C Dufour-Bouchard AA Trudeau C Using restrictive messages to limit high-fat foods or nonrestrictive messages to increase fruit and vegetable intake: what works better for postmenopausal women Eur J Clin Nutr 2010 64 194 202 19935818 10.1038/ejcn.2009.135

[bib30] Whybrow S Harrison CLS Mayer C Stubbs RJ Effects of added fruits and vegetables on dietary intakes and body weight in Scottish adults Br J Nutr 2006 95 496 503 16512935 10.1079/bjn20051489

[bib31] McMahon A-T Tapsell L Williams P Jobling J Baby leafy green vegetables: providing insight into an old problem? An exploratory qualitative study examining influences on their consumption Health Promotion J Austr 2013 24 68 71 10.1071/HE12901 23575593

[bib32] Tanumihardjo SA Valentine AR Zhang ZM Whigham LD Lai HCJ Atkinson RL Strategies to increase vegetable or reduce energy and fat intake induce weight loss in adults Exp Biol Med 2009 234 542 552 10.3181/0810-RM-293 19234056

[bib33] Rolls BJ Ello-Martin JA Tohill BC What can intervention studies tell us about the relationship between fruit and vegetable consumption and weight management Nutr Rev 2004 62 1 17 14995052 10.1111/j.1753-4887.2004.tb00001.x

[bib34] Epstein LH Carr KA Cavanaugh MD Paluch RA Bouton ME Long-term habituation to food in obese and nonobese women Am J Clin Nutr 2011 94 371 376 21593492 10.3945/ajcn.110.009035 PMC3142716

[bib35] Raynor HA Jeffery RW Phelan S Hill JO Wing RR Amount of food group variety consumed in the diet and long-term weight loss maintenance Obes Res 2005 13 883 890 15919842 10.1038/oby.2005.102

[bib36] Bucher T Van Der Horst K Siegrist M Improvement of meal composition by vegetable variety Public Health Nutr 2011 14 1357 1363 21557868 10.1017/S136898001100067X

[bib37] Turk MW Yang K Hravnak M Sereika SM Ewing LJ Burke LE Randomized clinical trials of weight loss maintenance a review J Cardiovasc Nursing 2009 24 58 80 10.1097/01.JCN.0000317471.58048.32 PMC2676575 19114803

[bib38] Alberti KGMM Eckel RH Grundy SM Zimmet PZ Cleeman JI Donato KA Harmonizing the metabolic syndrome: a joint interim statement of the international diabetes federation task force on epidemiology and prevention; National heart, lung, and blood institute; American heart association; World heart federation; International atherosclerosis society; And international association for the study of obesity Circulation 2009 120 1640 1645 19805654 10.1161/CIRCULATIONAHA.109.192644

[bib39] Grafenauer SJ Tapsell LC Beck EJ Batterham MJ Cluster analysis of clinical dietary data at the food group-level revealed greater weight loss was linked with altering dietary patterns characterised by non-core foods and drinks Eur J Clin Nutr 2013 67 330 336 23403877 10.1038/ejcn.2013.26

